# Epidemiological and clinical characteristics of severe poisonous mushroom poisoning in children in Yunnan Province: A retrospective analysis of 23 cases from 2020 to 2022

**DOI:** 10.1097/MD.0000000000043028

**Published:** 2025-07-04

**Authors:** Zhu Tian, Yang Li, Cheng Guo, Kai Liu, Juan Xie

**Affiliations:** a Comprehensive Pediatrics, Kunming Children’s Hospital and Kunming Medical University Affiliated Children’s Hospital, Kunming, Yunnan, China.

**Keywords:** ALT, blood purification, children, fatal poisoning, poisonous mushrooms, prognosis, PT

## Abstract

Poisonous mushroom poisoning is a common and highly fatal foodborne disease in southwest China. Children are at higher risk of severe illness and death due to limited hepatic reserve. However, studies on the poisonous mushroom poisoning in children are still limited. To analyze the epidemiological characteristics, clinical features, laboratory findings, treatment modalities, and prognostic factors of severe poisonous mushroom poisoning in children in Yunnan Province from 2020 to 2022. This was a retrospective study that included 23 hospitalized pediatric cases diagnosed with poisonous mushroom poisoning in Yunnan Province from 2020 to 2022. The Mann–Whitney U test was used to analyze the difference between alanine aminotransferase (ALT) and prothrombin time (PT) levels between the death and survival groups, and Spearman correlation analysis was used to assess their correlation with prognosis; the Fisher exact test and Fisher–Freeman–Halton test were used to compare the prognostic effects of blood purification treatment with different purification modalities. Poisoning cases clustered in summer-autumn, with *Amanita species* predominance. Acute liver injury type was the most common. ALT and PT levels were significantly higher in the death group (*P* = .013 and *P* = .025) and positively correlated with mortality (ALT: *R* = 0.538, *P* = .008; PT: *R* = 0.485, *P* = .019). Blood purification therapy significantly improved survival (64.7% vs 16.7%, *P* = .026), with no significant difference among modalities (*P* = .162). Elevated ALT and prolonged PT are key prognostic indicators of mortality in pediatric mushroom poisoning. Blood purification therapy improves survival. Dynamic monitoring of ALT and PT may aid early risk stratification and optimization of treatment strategies.

## 1. Introduction

Mushroom poisoning is a major public health concern globally, especially in countries where wild mushroom foraging culture is prevalent. According to the World Health Organization, tens of thousands of poisonings worldwide are caused by mushroom poisoning each year, with mortality rates ranging from 10 to 30%.^[1]^ Mushroom poisoning is frequent in some parts of China, particularly in Yunnan Province, due to the tradition of wild mushroom foraging. The region has a humid climate, rich biodiversity, and a wide variety of wild mushrooms. However, a general lack of recognition of toxic mushrooms by local residents, coupled with cultural practices driving foraging behavior, significantly increases the risk of poisoning.^[2]^ According to surveillance data from 2012–2023, China recorded 3229 mushroom poisoning cases resulting in 710 deaths. Among the 13 provincial-level regions that submitted laboratory confirmation results, Yunnan Province had the highest proportion of incidents with confirmed toxin detection.^[3]^ Other studies have also reported that Yunnan Province reported the highest number of wild mushroom poisoning incidents, cases, and deaths, accounting for 40.0%, 43.6%, and 41.0%, respectively, of the total number of poisoning incidents in the country.^[4]^ In addition, children are more susceptible to severe poisoning and progression to fatality due to insufficient physiological reserves and limited medical resources in the countryside.

Studies have shown that fatal poisoning by poisonous mushrooms mostly involves highly toxic species, such as *Amanita spp*, whose toxin α-amanitin causes apoptosis through inhibition of RNA polymerase II and mainly impairs liver and kidney function.^[5–7]^ Its toxic process is generally divided into 4 phases: latent phase, gastrointestinal phase, pseudo-healing phase, and organ damage phase.^[8,9]^ Severe patients tend to present with liver failure, coagulation disorders, and even multi-organ failure, and clinical staging is commonly characterized by acute liver injury type, rhabdomyolysis type, or both.^[10]^ Diagnosis relies on patient history, symptoms, and laboratory tests, especially liver and coagulation tests.^[11–14]^ Treatment is based on symptomatic support, hepatoprotection and enzyme lowering, and organ support.^[15]^ Blood purification therapies mainly include continuous renal replacement therapy (CRRT), plasma exchange (PE), and hemoperfusion (HP), all of which are considered to be effective for toxin removal.^[16]^

Despite the significant harm of poisonous mushroom poisoning to children, epidemiologic data on fatal mushroom poisoning in children in Yunnan Province are scarce, and the existing studies mainly focus on the adult population, with insufficient research on children.^[17]^ This study aimed to fill the gap in this field, systematically analyzing the epidemiological distribution, clinical manifestations, laboratory results, and treatment intervention effects of 23 fatal cases of poisonous mushroom poisoning in children in Yunnan Province between 2020 and 2022, exploring the key prognostic factors, and providing data support and strategy basis for the early identification and clinical treatment of this type of children.

## 2. Materials and methods

This retrospective study was approved by the Ethics Review Committee of Kunming Children’s Hospital (approval number: 2025-05-047-K01). The Ethics Committee exempted patients/guardians from written informed consent because the study was retrospective and data collection was anonymous.

This study employed a retrospective survey to collect fundamental Information on fatal poisonings of children attributed to poisonous mushrooms in Yunnan Province between 2020 and 2022 and contacted relevant hospitals to obtain specific medical records and reports. The hospitals included were all medical institutions in Yunnan Province that had reported cases of poisonous mushroom poisoning through the provincial disease surveillance system. The inclusion criteria included a clear history of wild mushroom consumption; having typical clinical manifestations of poisonous mushroom poisoning; laboratory test results supporting the diagnosis of poisoning; and having complete hospitalization records, treatment process, and outcome information (survival or death). All cases were uniformly reviewed by the research team to ensure diagnostic accuracy and data consistency. Exclusion criteria were the presence of other etiologies that could lead to liver or muscle damage, the presence of severe comorbidities or underlying diseases that affect prognosis, and lack of clear information on the type of fungus, or grossly incomplete medical records. A total of 24 cases of fungal poisoning in children were included, and 1 case was excluded due to incomplete medical records. Resulting in a total of 23 cases that met the criteria and were analyzed and compared according to the final survival outcome, divided into a death group and a survival group.

The following variables were collected in this study: age, gender, location, time of mushroom consumption, time of consultation, mushroom type, clinical characteristics, prognosis, laboratory results, and hemodialysis treatment modality. Among them, the types of poisonous mushrooms were determined on the basis of samples collected by family members or detailed descriptions and were categorized into 3 main groups: gooseberry genus (Amanita), helleborine-containing (Galerina) self-harvested mixed fungi and subnigricans. Clinical classification is based on the following criteria: Acute liver injury type: clinically significant elevation of alanine aminotransferase (ALT) levels, bilirubin abnormalities, and hepatic function abnormalities, but no markers of rhabdomyolysis; Rhabdomyolysis type: manifested by elevation of creatine kinase, myoglobinuria, and muscular symptoms, with no or very small elevation of liver enzymes; Both type: both liver injury and rhabdomyolysis. Prognosis was determined by hospitalization outcome (survival or death). Treatment modalities for blood purification included CRRT, PE, and HP, which were determined by the clinician based on the severity of the disease, the medical condition, and the specific modality to be used.

The study was statistically analyzed using IBM SPSS Statistics for Windows, Version 26.0 (IBM Corp., Armonk). Comparisons between groups were made using the Mann–Whitney *U* test to assess the differences between the dead group and the surviving group in laboratory indicators such as ALT, prothrombin time (PT), and activated partial thromboplastin time (APTT). Comparisons were made using the Fisher exact test for receiving or not receiving blood purification therapy; for the analysis of the association between blood purification modality and prognosis, the Fisher–Freeman–Halton exact test was used. Spearman rank correlation analysis was used to assess the correlation between laboratory indicators and the risk of death. All tests were 2-sided and the significance level was set at *P* < .05.

## 3. Results

### 3.1. Information on fatal poisoning by poisonous mushrooms (Fig. 1)

**Figure 1.**
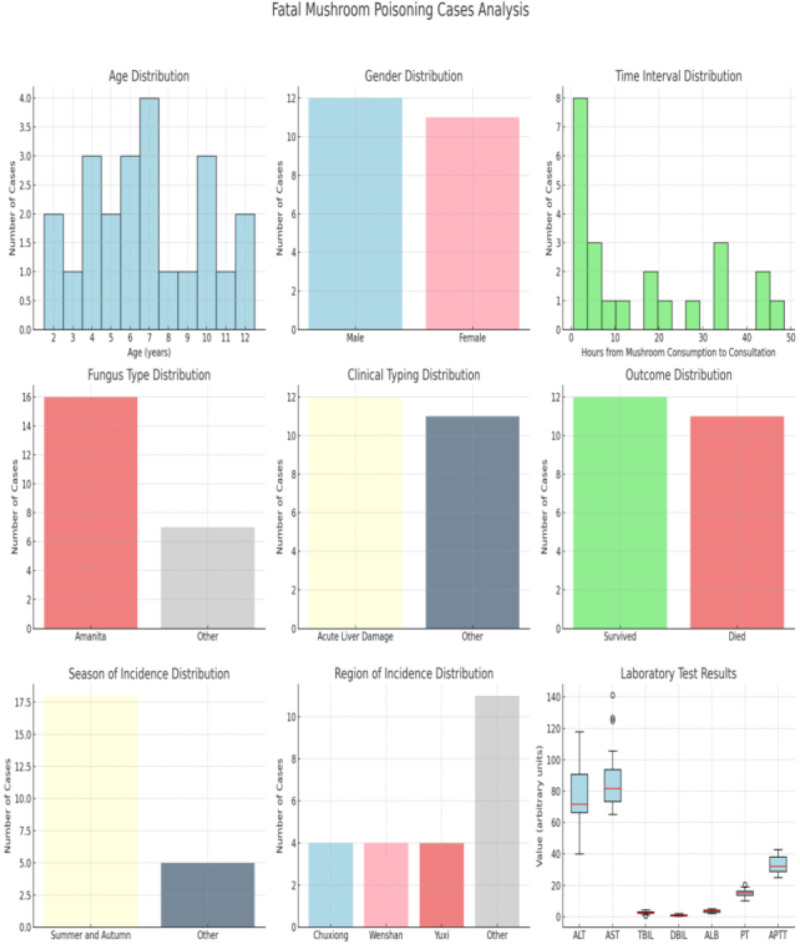
Basic characteristics of fatal cases of poisonous mushrooms.

Figure 1 depicts the basic characteristics of fatal mushroom poisoning cases including age, sex, location, time of consumption, time of hospital admission, type of mushroom, clinical classification, and prognosis. As can be seen from Figure 1, the age range of poisoning cases was from 2 to 12 years old, with males accounting for 52.2% and females for 47.8%. Chuxiong, Wenshan, and Yuxi cities accounted for 52.2% of the poisoning cases of poisonous mushroom poisoning. In the cases of poisonous mushroom poisoning, the time from consumption of poisonous mushrooms to hospital admission ranged from 0.5 to 72 hours, with an average interval of (14.3 ± 15.6) hours. Most of the poisoning cases of poisoned mushrooms involved *Amanita spp*, which accounted for 69.6% of the total number of cases. The predominant clinical phenotype of fatal poisonous mushroom cases was acute liver injury, which accounted for 52.2% of all cases. Other types accounted for 47.8% of the cases, including rhabdomyolysis type in 26.0% and both types in 21.8% of the cases. The prognosis of fatal poisonous mushroom cases was survival or death, with a survival rate of 52.2% and a mortality rate of 47.8%. The cases of poisonous mushroom poisoning in summer and fall accounted for 78.3% of the total number of cases. Among them, the highest number of catastrophic poisonings by poisonous mushrooms occurred in July-September, accounting for 43.5% of the actual problem. In the cases of poisoned mushroom poisoning, the levels of ALT, PT, and APTT were significantly increased.

### 3.2. Relationship between ALT, PT, APTT, and prognosis (Figs. 2 and 3)

**Figure 2.**
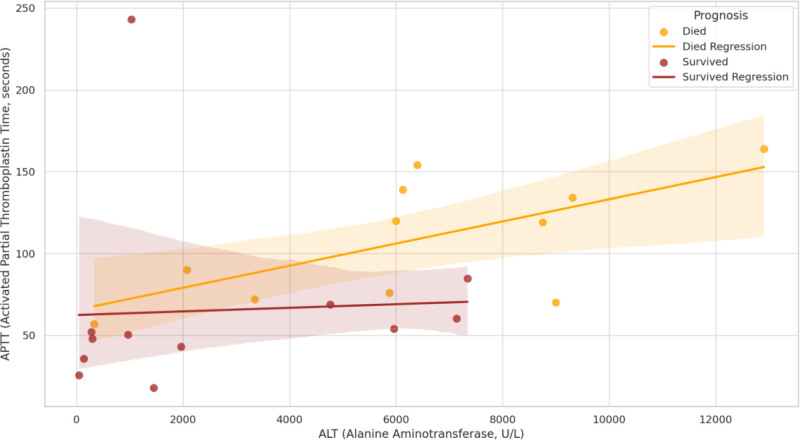
Relationship between ALT, APTT and prognosis. ALT = alanine aminotransferase, APTT = activated partial thromboplastin time.

**Figure 3.**
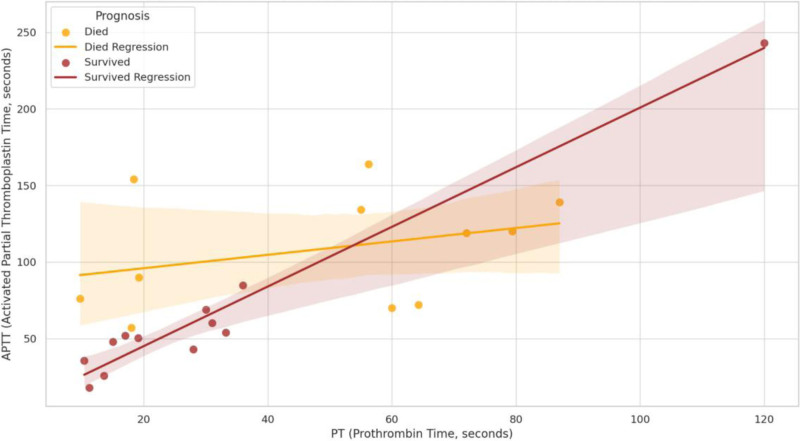
Relationship between PT, APTT and prognosis. APTT = activated partial thromboplastin time, PT = prothrombin time.

Scatter plots and their regression trend lines (95% confidence intervals) were plotted for the relationship between ALT and APTT, PT and APTT, respectively, categorized by prognosis into the death group (orange) and the survival group (brown), and 95% confidence intervals are shown. This result showed a positive correlation between ALT and APTT, PT, and APTT in both the death and survival groups (Figs. 2 and 3).

### 3.3. Relationship between PT, APTT, and prognosis (Fig. 4)

**Figure 4.**
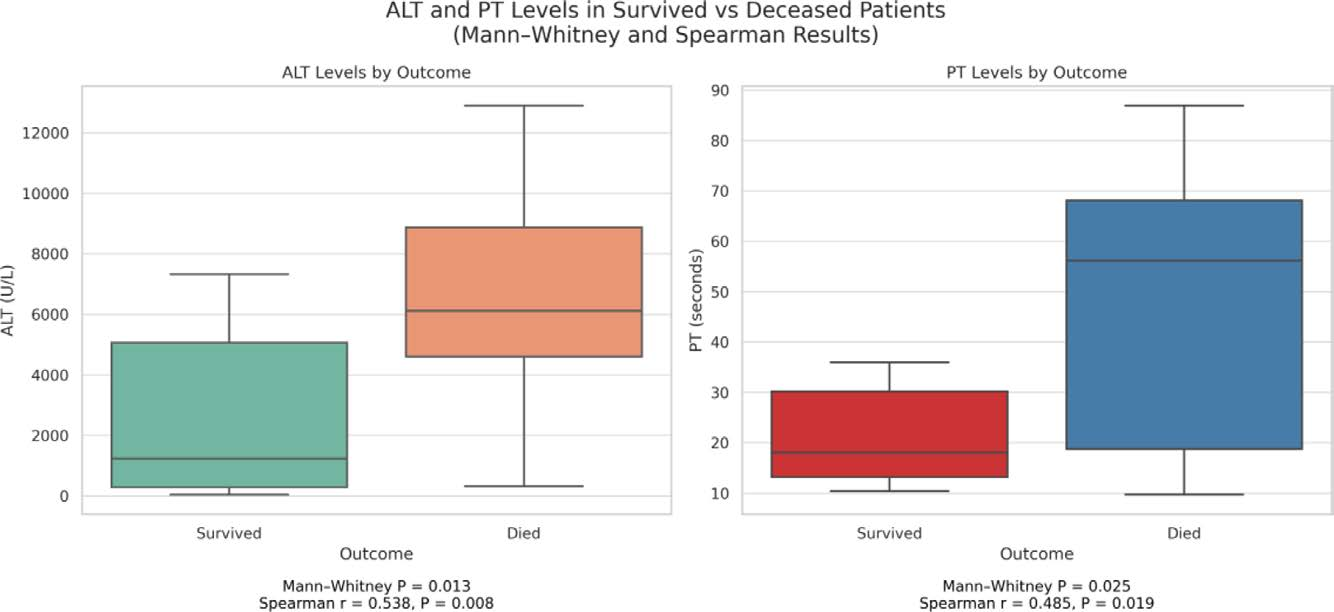
Relationship between ALT, PT and prognosis. ALT = alanine aminotransferase, PT = prothrombin time.

Figure 4 demonstrates the distribution of ALT and PT levels in surviving and deceased patients. According to the Mann–Whitney *U* test (*P* = .013 and *P* = .025, respectively), ALT and PT levels were significantly higher in the death group than in the survival group. In addition, Spearman correlation analysis showed that ALT and PT levels were moderately positively correlated with mortality (ALT: *R* = 0.538, *P* = .008; PT: *R* = 0.485, *P* = .019).

### 3.4. Relationship between blood purification treatment and prognosis in children with mushroom poisoning (Fig. 5)

**Figure 5.**
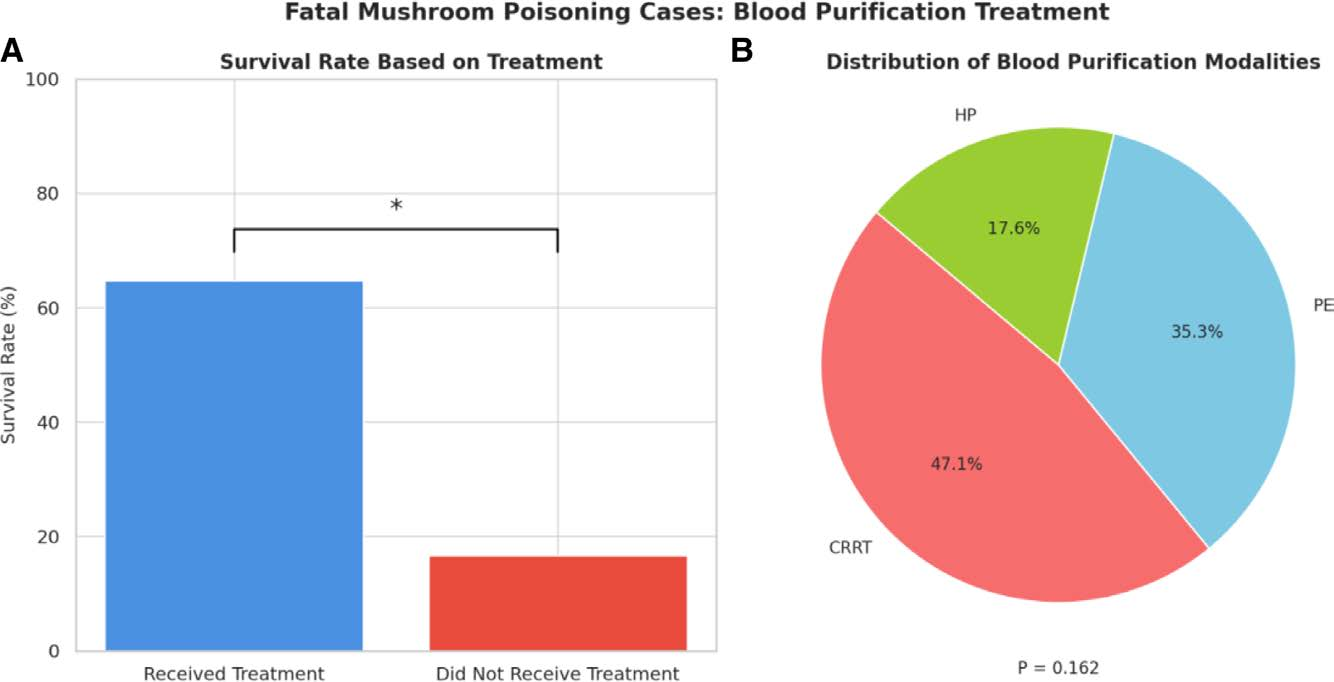
Relationship between blood purification therapy and prognosis in children with mushroom poisoning. CRRT = continuous renal replacement therapy, PE = plasma exchange, HP = hemoperfusion.

As shown in Figure 5A, the survival rate was markedly higher in children who received blood purification treatment (64.7%) compared with those who did not (16.7%). Statistical analysis using Fisher exact test revealed a trend toward significance (*P* = .026), suggesting that blood purification may be associated with improved survival. Figure 5B illustrates the distribution of different blood purification modalities among treated patients. CRRT was the most frequently used modality (47.1%), followed by PE (35.3%) and HP (17.6%). However, comparison of prognoses among the 3 modalities using the Fisher–Freeman–Halton exact test yielded no statistically significant difference (*P* = .162), indicating similar survival outcomes across methods in the current cohort.

## 4. Discussion

In this study, we retrospectively analyzed data on 23 cases of deaths from poisonous mushroom poisoning in children that occurred in Yunnan Province between 2020 and 2022 and investigated their epidemiological characteristics, clinical types, laboratory findings, hemodialysis treatment, and prognostic factors. Consistent with reports from other regions or countries, the present investigation showed that deaths from poisonous mushroom poisoning were concentrated in specific locations.^[18–20]^ This may be related to the lifestyle, economic conditions, knowledge of fungi, and dietary preferences of these populations and regions.

In this study, we found that both elevated ALT and prolonged PT were significantly associated with poor prognosis in children poisoned by poisonous mushrooms in Yunnan Province. ALT and PT represent liver injury and coagulation injury, respectively, and are of great value in determining the prognosis of poisonous mushroom poisoning. ALT, as an early biochemical marker of hepatocellular injury, is rapidly elevated after toxin inhibition of RNA polymerase II and induced hepatocellular necrosis, reflecting the occurrence and extent of liver injury, especially in children with poor liver reserve function.^[21–24]^ In contrast, PT prolongation usually occurs in the later stages of markedly impaired hepatic synthetic function, suggesting a decreased ability to synthesize coagulation factors, which is a manifestation of irreversible hepatic failure or severe coagulation disorders.^[25,26]^ This suggests that in clinical practice ALT can be used for risk assessment in the early stages of poisoning, whereas PT is more likely to be suitable for assessing late prognosis. In addition, we found that both ALT and PT levels were positively correlated with APTT, a trend that suggests that hepatocellular injury and coagulation disorders may coexist and synergistically aggravate during progression of the disease in children with poisonous mushroom poisoning. Therefore, the combined dynamic monitoring of the 2 may help to improve the sensitivity and timeliness of prognostic stratification and provide a basis for the timing of clinical intervention.

In the present study, the overall survival rate of poisonous mushroom poisoning in children was 52.2%, with acute liver injury type, which is basically consistent with previous reports in the literature both at home and abroad. For example, in 3 cases of collective poisoning triggered by gooseberry mushrooms reported by Sun et al, 4 out of 10 patients died, and all of the death groups showed more significant elevated liver enzymes and coagulation dysfunction and eventually died of acute liver failure.^[27]^ In a retrospective study of 455 patients with acute mushroom poisoning in Guizhou Province, it was also shown that the overall mortality rate was 10.33%, of which 89.36% had hepatic impairment and delayed treatment significantly increased the risk of death.^[28]^ In international studies, children accounted for 44% of the 41 cases of mushroom poisoning in Nepal, with a mortality rate of 29%, and in Turkey, children under 16 years of age accounted for 31% of the 294 cases, again with hepatic failure and coagulation disorders as the main mechanism of death.^[29–31]^ In addition, Erguven et al.‘s study on Amanita poisoning also confirmed that elevated ALT and prolonged PT were closely associated with poor prognosis.^[32]^ The present study not only verified the applicability of the above indicators in the pediatric population but also pointed out the possible time-phase value of ALT and PT in determining the severity, which provides a new idea for the dynamic assessment and stratified management of children with poisoning.

This study further evaluated the impact of blood purification therapy on the prognosis of children. The results showed that the survival rate of children who received blood purification therapy was significantly higher than that of those who did not, suggesting that this treatment has a positive effect in removing toxins from the body, maintaining the stability of the internal environment, and delaying organ failure.^[16]^ The mechanisms of different blood purification modalities have their own focus: CRRT removes small molecule toxins and metabolites through continuous slow filtration, which is suitable for hemodynamically unstable children; PE replaces medium and large molecule toxins in plasma, such as α-amanitin and improves coagulation dysfunction; and HP rapidly removes circulating toxins using adsorbent columns with rapid onset of action. Although each of these 3 treatment modalities has its own mechanistic advantages, this study did not find a statistical difference in improved survival, which may be related to the small sample size, the lack of combination of therapeutic modalities, and the differences in the children’s conditions. In addition, some children were unable to receive hemodialysis due to geographic or economic constraints, which may have introduced a certain selective bias. Therefore, clinical selection of purification modalities should be individualized according to the severity of the disease and the availability of resources, and timely intervention should be made at the early stage of intoxication as much as possible in order to optimize the therapeutic effect.

Overall, this study has certain novelty and practical significance. Firstly, unlike previous studies that mostly focused on adult poisoning patients, this study focused on 23 cases of poisonous mushroom poisoning in children in Yunnan Province and systematically analyzed their epidemiological characteristics, clinical manifestations, and prognosis, which fills the gap in the current research on pediatric severe poisoning. Secondly, the study population originated from a representative area with high incidence of poisonous mushroom poisoning in China, which has strong geographical adaptability and warning value. In addition, the results of the study clearly indicated that both ALT and PT levels are key indicators closely related to poor prognosis, and suggested that there may be complementary characteristics between them in the time course, which provides a new clinical idea for the dynamic risk assessment of poisonous mushroom poisoning. We suggest that dynamic joint monitoring of ALT and PT levels in children with poisonous mushroom poisoning can help to achieve early identification, precise stratification, and optimization of the timing of intervention in high-risk children, and improve the overall treatment effect.

Several limitations remain in this study. First, as a retrospective study, the data source relied on previous medical records and hospital reports, which may have incomplete information, record bias, and some key indicators missing, limiting the completeness and consistency of the data. Second, due to the small sample size (a total of 23 cases were included), some statistical results may be limited by efficacy and need to be further verified in a larger sample. Third, the multifactorial regression model was not established in this study, thus failing to adjust for potential confounders such as intoxication severity, age, and time from ingestion to consultation, which may affect the independent judgment of the relationship between ALT, PT, and prognosis. Fourth, some children who did not receive hemodialysis may have treatment selection bias due to economic or geographic limitations, which may also affect the accuracy of outcome comparisons. Finally, the source of cases in this study was limited to the Yunnan region, and the external generalizability of the results still needs to be further evaluated by multicenter studies.

## 5. Conclusion

This study retrospectively analyzed the data of 23 death cases of poisonous mushroom poisoning in children that occurred in Yunnan Province from 2020 to 2022. The results of the study showed that the fatal cases of poisonous mushroom poisoning in Yunnan Province were mainly concentrated in specific areas, the clinical manifestations were mainly of the acute liver injury type, elevated ALT levels and prolonged PT levels were risk factors for poor prognosis, and blood purification therapy played an important role in improving the survival rate. This study provides a reference for clinicians to develop effective preventive and therapeutic measures.

## Acknowledgments

We thank all the participants in this study, the various central hospitals for data support, and our colleagues who provided us with valuable advice and assistance during the course of the study. All those nominated for Acknowledgments in this paper have agreed to sign their names.

## Author contributions

**Formal analysis:** Zhu Tian, Yang Li.

**Methodology:** Kai Liu, Juan Xie.

**Software:** Cheng Guo.

**Writing – original draft:** Zhu Tian, Yang Li, Cheng Guo.

**Writing – review & editing:** Kai Liu, Juan Xie.

## References

[1] Saviuc P Danel V. New syndromes in mushroom poisoning. Toxicol Rev. 2006;25:199–209. 17192123 10.2165/00139709-200625030-00004

[2] Cheng J Cao Z Ma X. Mushroom poisoning: an overlooked cause of acute liver injury in China. Liver Int. 2017;37:468–9. 27943509 10.1111/liv.13334

[3] Yuan Y Zhang Y Zhou J. Detection and identification in reported mushroom poisoning incidents - China, 2012-2023. China CDC Wkly. 2024;6:1360–4. 39802087 10.46234/ccdcw2024.270 PMC11724132

[4] Li W Pires SM Liu Z. Mushroom poisoning outbreaks - China, 2010-2020. China CDC Wkly. 2021;3:518–22. 34594925 10.46234/ccdcw2021.134 PMC8393043

[5] Parasher A Aggrawal A. Prognosis and treatment options in cases of acute liver failure caused by mushroom poisoning due to Amanita phalloides. Int J Adv Med. 2020;7:875–80.

[6] Wu Z Li H Zhao W. Kidney toxicity and transcriptome analyses of male ICR mice acutely exposed to the mushroom toxin α-amanitin. Food Chem Toxicol. 2024;187:114622. 38531469 10.1016/j.fct.2024.114622

[7] Rodrigues DF Pires das Neves R Carvalho ATP. In vitro mechanistic studies on α-amanitin and its putative antidotes. Arch Toxicol. 2020;94:2061–78. 32193566 10.1007/s00204-020-02718-1

[8] Kubicka M Wilk J Dębiec P. Amanitine poisoning - cases, management, therapy results. J Educ Health Sport. 2023;13:276–80.

[9] Herink J. Review of present knowledge on the pathogenesis and pathophysiology of Amanita phalloides poisoning. Cas Lek Cesk. 1993;132:452–5. 8370055

[10] Chen Z Zhang P Zhang Z. Investigation and analysis of 102 mushroom poisoning cases in Southern China from 1994 to 2012. Fungal Divers . 2013;64:123–31.

[11] Li Y Mu M Yuan L. Challenges in the early diagnosis of patients with acute liver failure induced by amatoxin poisoning: two case reports. Medicine (Baltimore). 2018;97:e11288. 29979397 10.1097/MD.0000000000011288 PMC6076086

[12] Mohammed HMI Ahmad F. Mushroom poisoning: a rare etiology of acute liver failure. Cureus. 2023;15:e51144. 38283455 10.7759/cureus.51144 PMC10811487

[13] Roy S Saleem H. Mushroom poisoning and acute liver injury: a case-based review. Cureus. 2024;16:e75706. 39677988 10.7759/cureus.75706 PMC11646078

[14] Xu X Sun L Zhang Y. Poisonings caused by wild mushroom containing amanitin toxins - Shaoxing City, Zhejiang Province, China, 2019. China CDC Wkly. 2020;2:541–4. 34594698 10.46234/ccdcw2020.131 PMC8428458

[15] Garcia J Costa VM Bovolini A. An effective antidotal combination of polymyxin B and methylprednisolone for α-amanitin intoxication. Arch Toxicol. 2019;93:1449–63. 30891624 10.1007/s00204-019-02426-5

[16] Li Y Qiu Z Huang L. Extracorporeal membrane oxygenation combined with sequential blood purification in the treatment of myocardial damage and cardiac arrest caused by mushroom poisoning. Toxicon. 2021;197:65–9. 33872678 10.1016/j.toxicon.2021.04.011

[17] Li H Zhang H Zhang Y. Mushroom poisoning outbreaks - China, 2020. China CDC Wkly. 2021;3:41–5. 34594953 10.46234/ccdcw2021.014 PMC8392932

[18] Govorushko S Rezaee R Dumanov J. Poisoning associated with the use of mushrooms: a review of the global pattern and main characteristics. Food Chem Toxicol. 2019;128:267–79. 30995515 10.1016/j.fct.2019.04.016

[19] Karlson-Stiber C Persson H. Cytotoxic fungi: an overview. Toxicon. 2003;42:339–49. 14505933 10.1016/s0041-0101(03)00238-1

[20] Zhang L Chen QY Xiong SF. Mushroom poisoning outbreaks in Guizhou Province, China: a prediction study using SARIMA and prophet models. Sci Rep. 2023;13:22517. 38110518 10.1038/s41598-023-49095-0 PMC10728177

[21] Lin LY Tong YL Lu YQ. The characteristics of liver injury induced by Amanita and clinical value of α-amanitin detection. Hepatob Pancreat Dis. 2022;21:257–66. 10.1016/j.hbpd.2022.01.007 35168873

[22] Anbardar MH Soleimani N Kazemi K. Severe hepatotoxicity in mushroom poisoning by lepiota brunneoincarnata from complete recovery to liver transplantation: a case series with review on liver function tests and liver histopathology. Int J Hepatol. 2024;2024:2797712. 38288080 10.1155/2024/2797712 PMC10824578

[23] Mărginean CO Meliţ LE Mărginean MO. Mushroom intoxication, a fatal condition in Romanian children: two case reports. Medicine (Baltimore). 2019;98:e17574. 31593144 10.1097/MD.0000000000017574 PMC6799647

[24] Grama A Aldea C Burac L. Acute liver failure secondary to toxic exposure in children. Arch Med Sci. 2019;18:84–91. 35154529 10.5114/aoms.2019.87716 PMC8826985

[25] Lecot J Cellier M Courtois A. Cyclopeptide mushroom poisoning: a retrospective series of 204 patients. Basic Clin Pharmacol Toxicol. 2023;132:533–42. 36908014 10.1111/bcpt.13858

[26] Li S Liu K Liu Z. Effect of four-in-one optimized emergency nursing procedure on symptoms and vital signs of patients with mushroom poisoning. J Healthc Eng. 2022;2022:3387394. 35399847 10.1155/2022/3387394 PMC8989573

[27] Sun J Li HJ Zhang HS. Investigating and analyzing three cohorts of mushroom poisoning caused by Amanita exitialis in Yunnan, China. Hum Exp Toxicol. 2018;37:665–78. 28830233 10.1177/0960327117721960

[28] Xia C Lai F Wu J. Relationship between the start time of treatment and patient prognosis in cases of acute wild mushroom poisoning in a certain region of Guizhou Province, China from 2013 to 2020: a retrospective observational study and forecast. Hum Exp Toxicol. 2024;43:9603271241302192. 39624021 10.1177/09603271241302192

[29] Joshi A Awale P Shrestha A. Acute mushroom poisoning: a report of 41 cases. J Nepal Med Assoc. 2007;46:7–12. 17721556

[30] Eren SH Demirel Y Ugurlu S. Mushroom poisoning: retrospective analysis of 294 cases. Clinics (Sao Paulo). 2010;65:491–6. 20535367 10.1590/S1807-59322010000500006 PMC2882543

[31] Dadpour B Tajoddini S Rajabi M. Mushroom poisoning in the Northeast of Iran; a retrospective 6-Year epidemiologic study. Emerg (Tehran). 2017;5:e23. 28286830 PMC5325892

[32] Erguven M Yilmaz O Deveci M. Mushroom poisoning. Indian J Pediatr. 2007;74:847–52. 17901672 10.1007/s12098-007-0151-6

